# Experiences of Patients Undergoing Chemotherapy With Virtual Reality: Mixed Methods Feasibility Study

**DOI:** 10.2196/29579

**Published:** 2022-02-21

**Authors:** Anna Janssen, Jennifer Fletcher, Melanie Keep, Naseem Ahmadpour, Anika Rouf, Michael Marthick, Rebecca Booth

**Affiliations:** 1 Research in Implementation Science and eHealth Group Faculty of Medicine and Health The University of Sydney Sydney Australia; 2 Education Enterprise and Engagement The University of Sydney Sydney Australia; 3 Sydney School of Health Sciences Faculty of Medicine and Health The University of Sydney Sydney Australia; 4 School of Architecture, Design and Planning The University of Sydney Sydney Australia; 5 Faculty of Science The University of Sydney Sydney Australia; 6 Valion Health Sydney Australia; 7 Chris O'Brien Lifehouse Sydney Australia

**Keywords:** eHealth, digital health, virtual reality, cancer, chemotherapy, mixed methods research, virtual health, serious games, treatment

## Abstract

**Background:**

Current research into virtual reality (VR) use during chemotherapy shows that it can be an effective distraction intervention. However, there is limited research in adult patients and to investigate how VR can be sustainably implemented in health care organizations.

**Objective:**

The aim of this study was to explore the feasibility and acceptability of using VR for adult patients undergoing chemotherapy, and to identify the factors that would enable the sustained use of VR during chemotherapy in health care organizations.

**Methods:**

Patients undergoing chemotherapy were recruited to participate in a VR intervention during chemotherapy infusion. Participants were observed during the session and completed a postintervention survey. Each participant was invited to participate in a semistructured interview about their experience.

**Results:**

A total of 18 patients participated in the study, 5 of whom participated in semistructured interviews. Findings indicated that the use of VR was acceptable for patients undergoing chemotherapy and the intervention was also feasible. Some participants felt that the VR was an effective distraction during chemotherapy infusion, although most still seemed to be aware of how long their treatment was taking. Although VR was acceptable and feasible to patients, interviews identified several barriers to sustained implementation, including access to a reliable app library and impact on staff workloads.

**Conclusions:**

VR was acceptable to patients with a diagnosis of cancer undergoing chemotherapy treatment. Patients found VR beneficial for breaking up the monotony of treatment, to provide an additional choice of activity in addition to other recreation, and in some instances as a distraction from the treatment itself. However, there are challenges to address if VR is to be implemented in practice for this patient group.

## Introduction

### Background

Virtual reality (VR) describes the use of sophisticated hardware to generate virtual environments. Although VR has existed for several decades, the technology became particularly prominent in the mid-2010s with the release of affordable (from thousands of dollars to a few hundred dollars) commercially available VR head-mounted display (HMD) devices [1]. Since then, considerable innovation has occurred in the space, which has led to the development of a range of VR HMD solutions from high-end products that require the support of a powerful computer to less sophisticated VR solutions that can be powered by a smartphone [2].

The availability of affordable commercial VR hardware has paralleled a growing interest in the use of the technology in the health sector [3]. To date, VR has been used to support burn victims [4,5], as exposure therapy for phobias [6], in the management of traumatic brain injury [7], and to increase physical activity undertaken by members of the general public [2,8]. In addition to being used to support health care consumers, VR has been used to enhance medical training by augmenting existing approaches to surgical skills training [9], advanced life support training [10], and for developing empathy skills [11].

In the context of cancer care, there is a small but growing body of research into the use of VR technology, primarily in the pediatric setting. There is literature exploring the use of VR as a distraction from anxiety in children during painful procedures related to cancer treatment or its side effects [12,13]. This research extends the literature on mechanisms for distracting patients undergoing chemotherapy, such as music therapy [12], social media use, and reading [13]. Research to date has shown that active distractions can be more effective than passive distractions for pediatric patients [14] and in adult populations [15]. The immersion caused by VR creates a more active distraction than other mechanisms, which may be effective for patients undergoing certain procedures. For cancer patients, VR has been shown to be more effective than other forms of distraction for relieving anxiety, depression, and fatigue during chemotherapy [12].

There is a small body of research indicating that VR can distort the perception of time during clinical procedures. One study showed that patients undergoing chemotherapy for breast and colon cancer perceived the procedure as being shorter than it was in reality [16]. Although not in the context of cancer care, a study of dental patients found that VR distorted the perception of time, with patients reporting that the procedure they were undergoing took less time than it actually did [17]. A recent systematic review evaluated the evidence supporting the use of VR among patients in acute inpatient medical settings, and found only two studies that reported findings related to the temporal distortion effect of VR [18]. However, it should be noted that in the context of those two studies, the temporal distortion measured was a change in time thinking about pain while using VR, which is distinct from measuring a change in time a person perceives to have passed due to the use of VR. This would suggest that temporal distortion relating to the change in time a person perceives to have passed because of VR-induced distraction is an underexplored area of research. Addressing this gap in the context of cancer care may be advantageous because there are some groups of patients who report greater satisfaction with treatment if they perceive their chemotherapy duration to be shorter than it is [19]. Other areas where there may be benefits of further researching temporal distraction from VR include certain pediatric groups such as those undergoing painful dental procedures, where greater levels of distraction can improve patient rapport and reduce disruptive behavior [14].

The role of serious games as a distraction technique during chemotherapy and to improve experiences of care in cancer patients has also been explored in the literature. A recent review of serious games exploring whether they can positively impact children with cancer found that such research has largely focused on the use of games in three categories: education, motivation, and distraction [20]. In adult cancer patients, serious games have been shown to be effective for improving patient self-management of nausea resulting from chemotherapy [21]. Researchers have also explored the role of serious games to improve the quality of life for breast cancer patients undergoing chemotherapy, demonstrating that patients are generally satisfied with the use of serious games for this purpose [22]. However, to date, there has been less research undertaken on the use of serious games via VR with adult cancer patients undergoing chemotherapy, or the use of other types of VR apps with this cohort.

Additionally, researchers have explored the use of VR to support psychological well-being in children hospitalized with cancer [23]. A smaller number of studies have been undertaken on the use of VR with adult cancer patients. There is some research to suggest that VR may be a cost-effective distraction technique for patients undergoing chemotherapy infusion [16], and that VR may help manage the adverse effects of chemotherapy infusion in older women with breast cancer [24]. A recent mini-review of the use of VR with patients undergoing chemotherapy concluded that there were several gaps in the research in this area, including limited research on the potential adverse events of using VR during chemotherapy infusion and a lack of research into the barriers to implementing VR in practice [12].

Patients with cancer are a notably diverse group. This is because “cancer” describes a group of over 100 different diseases that involve the inappropriate proliferation of cells [25]. Cancers can be diagnosed in pediatric, adolescent, young adult, and adult individuals, and the prevalence of different cancers can also vary according to genetic background and geographic region [26,27]. Medical interventions vary considerably depending on the diagnosis and the prognosis. Interventions include systemic therapies such as chemotherapy and immunotherapy, surgical interventions, and radiation therapy. Cancer patients can be receiving medical interventions for different reasons, including to prevent disease recurrence or palliatively to manage symptoms of the disease [28,29]. Systemic therapies can be delivered as an outpatient therapy where appropriate, which means that patients must go to the hospital for a scheduled appointment to receive treatment but are not admitted to hospital.

In light of this diversity, patient-centered VR interventions for cancer patients should be flexible or appropriately varied to respond to the needs and interests of patient groups [30]. It has been noted in the literature that there is limited research seeking to understand the characteristics of patients that will gain the greatest benefit from immersive technology such as VR [31,32]. This would enable a more personalized “prescription” of VR interventions to patients for whom it would be most useful. More recent research has noted the importance of considering patient characteristics such as motor, visual, or vestibular impairments in the design and implementation of VR for patients with acquired brain injuries, as lack of such consideration could be a barrier to the use of VR by this group [33].

In addition to the limited understanding of the characteristics of patients who will likely respond well to VR interventions, there is limited understanding of the VR interventions that are suitable for, and of interest to, patients with chronic conditions [34]. Understanding what makes VR acceptable and feasible to patients with chronic conditions may be used to inform decisions around which features should be prioritized in different applications. For cancer patients, this gap is particularly problematic because there are specific limitations on the use of VR with this group, including a potential need to operate the device with one hand to avoid interfering with drips or cannulas, and ease of learning the game mechanics and controls due to varied experiences with VR or video games.

### Objectives

The aim of this study was to explore the feasibility and acceptability of using VR to improve the quality of life of adult patients undergoing chemotherapy, and to identify the factors that would enable the sustained use of VR during chemotherapy in health care organizations.

The following research questions were addressed: How feasible is the use of VR for patients undergoing chemotherapy? How acceptable is the use of VR for patients undergoing chemotherapy? How distracting is VR for patients undergoing chemotherapy? How challenging would it be to implement VR in routine clinical practice?

## Methods

### Study Design

This study used a mixed methodology with observations, followed by online surveys, semistructured interviews, and analysis of uptake metrics for the intervention. The feasibility of the intervention was primarily assessed via observation data. The acceptability was primarily assessed via postintervention survey data and interview data. The extent to which VR was an effective distraction during chemotherapy was assessed through data collected from the postintervention survey and interviews. The implementation barriers and enablers were primarily assessed via interview data, along with some observation data.

### Setting and Participants

The study was undertaken at a large metropolitan health care organization specializing in care for patients with a diagnosis of cancer. The health care organization has a day therapy suite to deliver chemotherapy and immunotherapy to cancer patients. Patients undergoing chemotherapy visit the day therapy suite to have their scheduled treatment. The length of the treatment on an individual day can vary considerably depending on the patient’s protocol, but most patients generally have at least 1 hour of treatment. At the start of a chemotherapy session, patients are usually cannulated, injected with their chemotherapy dose, receive an infusion of the required drugs until the end of their therapy session, and have their lines flushed before removing the cannula. Prior to the session, patients often have blood tests and await results, which means they have often already spent a substantial amount of time on site before treatment.

Inclusion criteria for the study required potential participants to be patients 18 years old or over with a histologically confirmed diagnosis of cancer, who were undergoing chemotherapy. The exclusion criterion for the study was that potential participants could not be part of a commercial phase I trial.

A member of the health care team at the intervention site approached patients and provided them with an Expression of Interest (EOI) form and a copy of the Participant Information Statement and Consent Form. If patients were interested in participating in the study, they shared their contact details via the EOI form. A member of the research team would telephone the potential participants to explain the study further, answer questions, and identify a suitable chemotherapy session for receiving the VR intervention. Participants provided written consent prior to participating in the intervention.

### Procedure

**Figure 1:**
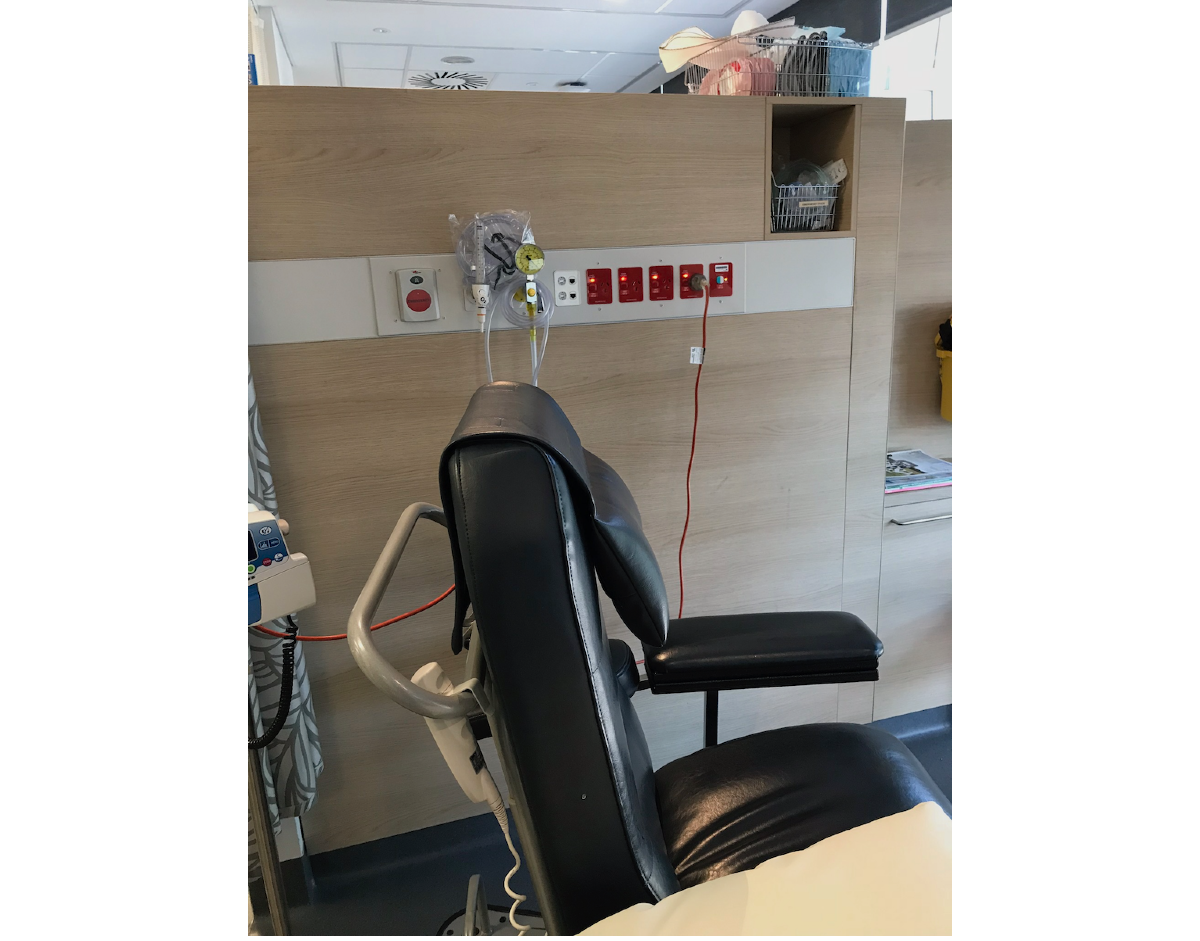
Image of the location where the participants undertook their virtual reality sessions.

**Figure 2:**
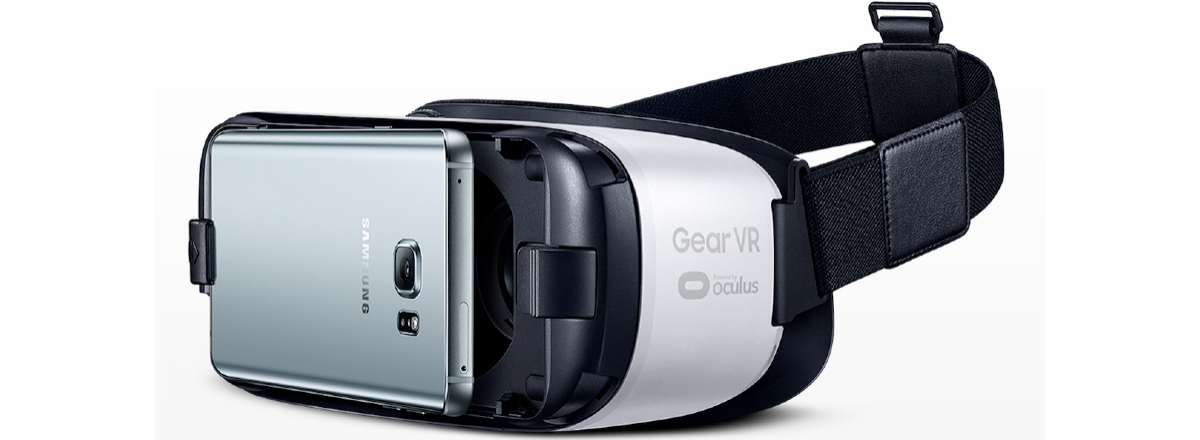
The Gear VR headset used in the study.

The intervention was delivered using the Samsung Gear VR HMD headset. The VR hardware was chosen as the study site had previously invested in the technology and wanted to further understand the feasibility of implementing it in practice. Each participant only undertook a VR session once during the study. Figure 1 shows an image of the study site and Figure 2 shows an image of the Gear VR headset.

Once participants had been set up with their chemotherapy infusion, a member of the research team provided them with the VR headset and a brief overview of how the hardware worked, how to navigate the interface, and the apps that were available to use during the intervention. Participants then completed a brief survey on their previous experience with digital games and VR.

Upon completion of the survey, participants selected an app they would like to play during their chemotherapy appointment from a list of 10 apps. The researcher was available for support while the participant was engaged in VR. Participants could choose to play with more than one VR app during their chemotherapy appointment, although they were limited by the overall time of the appointment. If they chose to play additional VR app(s) during their appointment, the researcher would assist them in selection.

To measure the feasibility of the intervention, usage behaviors were captured during the intervention. A researcher observed participants during the intervention and took notes. Data collected through observation notes included apparent ease of the use of controls, duration of the VR session, number of apps the participant chose to use during the intervention, and comments about the experience.

Upon completion of the VR intervention, participants completed a postintervention survey (see Multimedia Appendix 1) informed by the Player Experience of Needs Satisfaction (PENS) instrument [8] and an adverse event checklist that allowed them to visually indicate any adverse events experienced (see Multimedia Appendix 2). The postintervention survey consisted of nine items about the VR experience, including eight items ranked on a 1-7 Likert scale and one yes-or-no question. In addition to the survey, participants completed the Edmonton Symptom Assessment System (ESAS-r) questionnaire postintervention. The ESAS-r describes the individual’s symptom severity based on a scale ranging from 1 to 10 to quantify the level of distress in cancer patients for nine common symptoms of the disease [35]. Responses were averaged and higher scores indicated higher levels of distress. The ESAS-r has high internal consistency, convergent validity, and construct validity (α=.87, .84, and .82, respectively [36]), and has been used to monitor symptoms of distress in a range of contexts, including cancer, palliative care, and nephrology [37].

Participants were invited to participate in semistructured interviews 4-6 weeks after the intervention. Interviews were not undertaken directly after the intervention so as to give the participants some time to reflect on their experience prior to the interview, enabling them to provide richer responses on the benefits and disadvantages of VR for individuals undergoing chemotherapy. These interviews explored participants’ experience of the VR intervention. The interviews were conducted over the phone, took between 15 and 20 minutes, and were conducted by a different researcher from the researcher who administered the VR intervention. The interview schedule was designed to gain a high-level understanding of the participants’ experiences with the VR intervention, identify challenges they encountered, or indicate opportunities for improvement in future. Questions in the interview included: How was your experience with VR during the study? How effective did you find the VR for reducing your anxiety about treatment? Were there any benefits you experienced from the use of VR during treatment? Were there any concerns or issues you had with the use of VR during your treatment? Interviews were audio-recorded, transcribed by an external company, deidentified, and analyzed.

### Data Analysis

Descriptive statistics of quantitative data were analyzed using IBM SPSS 24.0. Due to the small sample size of this feasibility study, there was not sufficient power to conduct inferential statistical analyses.

Observation and semistructured data were reviewed using content analysis. Interview transcripts and observation notes were read to obtain an overall sense of the themes, and then the content was grouped into categories related to the research questions, or into new categories if the data did not align with any of the existing categories. Categorization of transcript and observation data was discussed by two authors (AJ and JF) to align each researcher’s categorization. Fully analyzed data were grouped in response to the research questions.

### Statement of Ethics

This research project received ethics approval from Sydney Local Health District Human Research Ethics Committee (protocol number X18-0313).

## Results

### Demographics

A total of 19 individuals participated in the VR intervention. One participant was unable to complete the intervention due to technical issues with the VR hardware. As such, only 18 participants, 10 men and 8 women, completed both pre- and postintervention surveys and the intervention, 5 of whom participated in a follow-up interview. Presurvey data for the participant who did not receive the intervention are included in the results presented herein, whereas no observational or postintervention data from this participant were included in this analysis.

Of the 18 participants, the majority (n=14, 78%) had no experience with VR, and all felt inexperienced using the technology (n=18, 100%). Several participants had no experience playing video games (n=6, 33%), although more of the participants had experience playing video games than in using VR. The majority of participants (n=12, 67%) had no experience playing smartphone games. Of the remaining six participants who had experience playing smartphone games, two were different from the six who had experience playing digital games.

### Feasibility of VR for Patients Undergoing Chemotherapy

During the VR intervention, participants were given a choice of which apps and the number of apps they played. The apps used in the intervention were shortlisted from the Occulus store based on rating, price, genre, comfort, and potential to play one-handed. After shortlisting based on this information, all games were play-tested to evaluate their functionality and confirm the ability to play one-handed (required for patients undergoing chemotherapy treatment). The majority of participants opted to play both game apps and experience apps. However, two participants only played apps that were experiences and three participants only played apps that were games. Table 1 provides a list of the apps the participants could choose from during the intervention.

Observations during the VR sessions showed that some of the issues that the researcher anticipated would make the intervention potentially unfeasible were actually less problematic than expected. The researcher anticipated some difficulty with adjusting the headset due to the headwear of the participants, and therefore explained what the headgear would be like prior to the session. All participants seemed to be at ease with adjusting their headwear and were happy to remove any scarves or hairpieces for the session. Two participants struggled to find an ideal focus for the lenses and reported that it was slightly blurry at times. The hand controller was used to navigate through games. For most participants, it was easy to use; however, occasionally, it did need to be reset by the researcher because its “direction” function went out of synchronization. The “direction” function going out of synchronization typically made the cursor appear in spots that were not directed by the participant or would disappear off the user screen.

One participant was wearing cooling mitts (hand coverings with ice packs) but could still use the VR hand controller with ease, inside the mitts. For the sessions, the researcher requested that participants be cannulated in their nondominant arms. Two participants did use the VR controller with the hand of the arm they were cannulated in but reported that this was still comfortable for them. The selected games did not require any wild arm movements.

Analysis of postsurvey responses indicated that the majority of participants did not experience motion sickness (14/18, 78%). The researcher observed that in the four cases of reported motion sickness, the feeling was mild and only one participant asked to change the game due to the sensation.

**Table 1:** Apps patients could choose from during the virtual reality intervention.

App Name	Description	Publisher	Date published	App type
Sphæres	A series of cinematic virtual reality experiences to improve mindfulness and help with relaxation	Atmosphæres	November 2018	Experience
Smash Hit VR	An abstract game where a sphere is shot at glass objects in order to progress the level and get a high score	Mediocre	September 2015	Game
Invasion	An animated film about two aliens who try to take over Earth, but are thwarted by two bunnies	Baobab Studios Inc	March 2016	Experience (short film)
Meeting Rembrandt	An immersive experience for exploring the world of the Renaissance painter Rembrandt van Rijn in his studio as he creates the painting the Night Watch	Oculus Studios	September 2017	Experience (short film)
Ocean Rift	An aquatic safari park that enables exploration of an underwater world	Picselica Ltd	March 2015	Experience
Bait	A fishing game where the goal is to catch a rare fish to save the local aquarium	Resolution Games	March 2016	Game
Espr	A puzzle game where psychic abilities are used to solve challenges	Coatsink	May 2015	Game
Happy Place	A virtual environment that promotes positive emotions and calmness at a lakeside campsite in the mountains	Mimerse	October 2016	Experience
Forest of Serenity	A guided tour through a forest populated by exotic plants and animals, narrated by Sir David Attenborough	Holosphere VR	July 2018	Experience
Zen Garden	A relaxing environment that uses musical instruments controlled by the player’s gaze to encourage calm and tranquility	Carry Castle AB	November 2016	Experience

### Acceptability of VR for Patients Undergoing Chemotherapy

#### Experience with VR During Chemotherapy

The majority of participants (14/18, 78%) indicated that they found the VR intervention enjoyable by giving a ranking of at least 5 out of 7. Most participants (15/18, 83%) indicated they would use VR again. Table 2 highlights the participant responses to survey questions 2-9.

**Table 2:** Participant responses to survey questions 2-9.^a^

Participant	Enjoyed activity	Activity fun	Activity boring	Activity did not hold my attention	Activity was interesting	Activity was enjoyable	I thought about enjoying the activity while I did it	I would try virtual reality again
VR001	5	4	2	2	6	5	5	6
VR002	6	6	1	1	5	6	6	7
VR003	7	7	1	1	7	7	4	7
VR004	7	7	1	1	6	7	7	7
VR005	6	7	1	1	4	7	7	7
VR006	6	6	1	2	6	6	2	7
VR007	7	7	1	1	7	7	6	1
VR008	3	3	3	2	4	4	3	4
VR009	7	7	1	1	7	7	7	7
VR010	4	4	3	3	4	4	5	4
VR011	7	7	1	1	7	7	7	7
VR012	7	7	1	1	7	7	7	7
VR013	3	5	1	1	5	6	5	6
VR015	3	3	1	1	4	3	3	7
VR016	7	7	1	1	7	7	5	7
VR017	7	6	1	1	7	7	5	7
VR018	6	6	1	1	7	7	6	7
VR019	5	6	1	1	5	7	4	7

^a^The survey asked participants to indicate a response on a 1-7 scale, where 7 was strongly agree and 1 was strongly disagree.

#### Recreation During Appointments

Participants reported reading, working on laptops, and playing on smartphones during their other chemotherapy sessions as forms of recreation they engaged in during appointments. Several participants stated that they sometimes sleep through the chemotherapy and some were accompanied by a family member or friend.

Participants laughed, smiled, and conversed about the scenery in the VR intervention, the rules, and game play while engaged in VR. Participants reported the experiences as relaxing and the games to be fun. Some participants liked the competition aspect of the games and were reluctant to stop (even at the end of their chemotherapy session).

When interviewees were asked about their experiences with VR as an alternative recreation during chemotherapy, the majority were positive about the experience. The reasons the VR intervention was considered to be engaging for interviewees varied. Several interviewees noted that chemotherapy was boring and that VR provided a good distraction from the monotony: “...that’s why it would be quite useful to have the VR because you get a bit bored.” [Participant VR003]

Several interviewees indicated that having chemotherapy can cause a lot of anxiety, and depending on the appointment, it can be more or less difficult to show up. One interviewee noted that the idea of the VR session motivated them to attend their chemotherapy appointment, when they previously were anxious about going.

…I was like, “I can totally do this chemo session now.” From that perspective it [VR] was brilliant, I definitely was looking forward to it. I remember when I walked in, I said “are we doing it?” It definitely made the experience. It made it so much easier to go into it and I was so looking forward to it. It was all I talked about.

Participant VR007

By far the most consistent feedback from interviewees was that they liked having the choice of VR during chemotherapy. While many interviewees felt they would continue to use other forms of recreation during their appointments, the idea that VR was an option was very appealing: “…Look, it’s just another option there for us...The fact that there’s a choice, I think that’s always a good thing.” [Participant VR004]

#### Experience With Hardware

Overall, interviewees found the VR hardware relatively easy to use and not uncomfortable to wear during their chemotherapy appointment.

…I don’t remember it being a problem. I think once we sit it on my head properly, it was much better. But yeah, it was perfectly lightweight and all good to go. It was interesting. I’ve never used virtual reality before, but I really enjoyed it.

Participant VR007

Some participants did mention that there were some issues with the fit of the headset, and that it could slide off the head during some activities.

…With the headset and all that, when you’re sitting there and someone says something to you and you reach down to do something. It comes off…because it’s an ordinary iPhone, we were having problems.

Participant VR002

One interviewee commented that the VR hand controller was not reliable or user-friendly. Participants were provided a handheld controller accessory they could use to navigate the apps. However, the apps did not require the use of this controller and participants could use the touch controls on the headset if they chose.

Several interviewees gave feedback about aspects of the VR operating system itself. One set of issues focused on aspects of the system such as inability to adjust the lens focus, which led to blurry images and resulted in limited immersion within the VR world. Several interviewees also reported that the home screen of the VR product was difficult to navigate, which was a challenge for launching individual apps.

#### Patients’ Perspectives of the VR Apps

Observations during the VR sessions suggested that participants were enthusiastic to try out several different apps to get an overall feeling of the range of VR capabilities. The majority of participants who tried a relaxation app got the idea of the app fairly quickly and then wanted to also try a game-style app. During the relaxation apps, participants generally relaxed, turning their heads to see more of the scenery, and some describing the setting they were in. Several asked to stop the app because they were going to fall asleep.

During gaming apps, the mood of the participants seemed to be quickly elevated, indicated by participants laughing and smiling, and making comments about what was going on. Many participants wanted to know what the highest score of the previous participant was (so they could try to beat it).

Interviewees described playing a considerable variety of apps during the VR intervention. Several interviewees commented on the amount of choice being a positive aspect of the experience, although most interviewees preferred using game-based rather than experience-based VR apps. Experience-based VR apps did not have any overt rules or game mechanics, and included activities such as guided meditation. Participants reported that games were preferred because they allowed the participant to actively do something, whereas experiences could make it difficult to disconnect from the busy clinical environment.

…I prefer gaming things. I prefer doing things. I prefer keeping busy personally, rather than sort of just lying back and I think maybe if you relaxed or concentrated too much, you’d focus too much on the chemotherapy.

Participant VR001

### VR as a Distraction for Patients Undergoing Chemotherapy

#### Distraction and Temporal Distortion

Participant VR sessions ranged from 7 to 68 minutes (mean 29.9, SD 17.8). When asked to approximate how long they thought they spent in the VR sessions directly after completing the session, participants’ perception of the session duration ranged from 8 to 90 minutes (mean 35.6, SD 22.4). The majority of participants estimated the duration spent in the VR session fairly accurately (mean 5.12, SD 3.8). Table 3 provides an overview of participant time spent in the VR intervention, mapped against estimated time spent in the intervention.

During the VR sessions, participants seemed very aware of how long their treatments take, and therefore were able to accurately estimate how long they spent in the VR session. For the majority of participants, they enjoyed the experience so much that they were engaged with VR for their entire treatment session.

One participant requested before the session that the VR be playing during cannulation because they find the cannulation and drug administration to be very painful. The clinical nurse specialist allowed the researcher to set up the VR first. The participant found the VR experience to completely distract from the pain. Interestingly, each time the participant came out of an app to try another, they reported that they suddenly noticed the pain. This participant noted that the VR was a complete distraction from the strong pain he experiences during treatment. For two different participants, an accompanying visitor mentioned to the researcher that the participant was having a particularly bad day and that their mood had picked up substantially.

**Table 3:** Overview of participant time spent in the virtual reality intervention, mapped against estimated time spent in the intervention.

Participant	Actual intervention time (minutes)	Perceived intervention time (minutes)	Difference
VR001	34	30	+4
VR002	45	23	+22
VR003	90	55	+35
VR004	30	15	+15
VR005	30	28	+2
VR006	35	36	–1
VR007	15	15	0
VR008	20	14	+6
VR009	15	36	–23
VR0010	8	14	–6
VR0011	60	62	–2
VR0012	40	16	+24
VR0013	7	7	0
VR0015	60	—^a^	—
VR0016	15	18	–3
VR0017	40	34	+6
VR0018	—	21	—
VR0019	60	68	–8

^a^Missing data, which occurred because of unanticipated interruptions to the intervention session for the individual participants, leading to inaccuracies in collection of observation data or inability to collect data.

#### Well-being and Physical Health

Participants reported low scores for each of the nine symptoms of cancer on the ESAS-r: pain (mean 1.22, SD 2.42), tiredness (mean 2.11, SD 2.52), drowsiness (mean 2.06, SD 2.44), nausea (mean 0.61, SD 1.20), lack of appetite (mean 0.76, SD 1.95), shortness of breath (mean 0.22, SD 0.55), depression (mean 0.72, SD 1.74), anxiety (mean 1.11, SD 2.08), and well-being (mean 2.41, SD 2.60). Table 4 shows the participant scores for each ESAS-r domain.

Participants were instructed to provide feedback on any physical discomfort they had experienced during the intervention to ensure they did not require any support from a cancer care nurse. No participants experienced physical discomfort as a result of the intervention. Two participants indicated pain from the site where the chemotherapy intravenous infusion was on their arm.

**Table 4:** Participant responses to the Edmonton Symptom Assessment System (ESAS-r) scale.^a^

Participant	Pain	Tiredness	Drowsiness	Nausea	Lack of appetite	Shortness of breath	Depression	Anxiety	Well-being
VR001	4	2	1	3	UA^b^	2	1	0	5
VR002	0	0	0	0	0	0	0	0	0
VR003	0	3	3	0	0	0	0	0	4
VR004	1	1	1	0	0	0	0	1	—^c^
VR005	0	5	5	0	1	0	0	2	1
VR006	5	8	5	1	1	1	7	7	8
VR007	0	5	5	0	0	0	0	5	2
VR008	0	0	0	0	0	0	0	0	0
VR009	1	0	0	0	0	0	1	0	1
VR010	0	5	5	0	8	0	0	0	5
VR011	0	0	0	0	0	0	0	0	0
VR012	0	0	0	0	0	0	0	0	0
VR013	0	0	0	4	0	0	0	0	5
VR015	0	0	0	0	0	0	0	0	0
VR016	1	1	1	1	1	1	1	1	1
VR017	9	5	7	0	0	0	0	0	6
VR018	0	0	0	0	0	0	0	0	0
VR019	1	3	4	2	2	0	3	4	3

^a^The questionnaire asks participants to rate cancer symptoms on a 0-10 scale where 10 indicates the worst distress and 0 the least distress.

^b^UA: unanswered.

^c^Missing data, which were excluded for the calculation of well-being but response data were included for the other eight symptoms. Pairwise deletion was used to calculate means and SDs, which was only necessary for the missing well-being score for one participant.

### Barriers and Enablers of Implementing VR in a Day Therapy Suite

#### Technological Literacy

Participants reported concern that, being “older,” they would find it difficult to use the controls, but this did not eventuate. Other participants were concerned about falling asleep with the headset on because the experiences were very relaxing, and that they would likely need practice at setting up before they could negotiate doing it on their own in the day therapy suite.

#### App Choice

As mentioned above, participants highlighted that having a choice of recreational activities during an appointment was very appealing, and the variety of VR apps was an enabler to sustained use. Participants’ feedback highlighted the need for a wider variety of apps available to patients, but acknowledged that curating this content could be difficult. Participants also stressed that the apps would need to be reliable (nonglitchy) and having more choice would exacerbate this issue.

#### Hardware Usability and Need for Technical Support

Staff workload was identified as a factor influencing the sustained use of VR during chemotherapy. Interviewees noted that many patients would need some support learning to navigate the platform, and without a researcher present, this burden would fall on staff. One participant enjoyed the experience so much, she said she would gladly be a volunteer to set chemotherapy patients up with games in the future. They also mentioned that many chemotherapy facilities have volunteer groups that assist with recreational activities.

…I think it’s a really good idea [VR]. The only setback I see is something that may possibly occur, the nursing staff having to deal with anything with the machinery, because they’re incredibly busy and to then have to come back because something’s not working to some technical things, not working might be problem for them. And also infection control issue with the machinery, so how it’s cleaned afterwards and how it is maintained?

Participant VR001

…I think you definitely would need to have someone there to troubleshoot. Most anyone 40 and under, all have phones and know how things work and there’s always a back button and there’s always a click on this and then that takes you somewhere. That sort of stuff is really simple. But yeah, it’s the setting up originally like connecting it to the headset. Connecting it to the phone I guess, because the phone is just in the headset and connecting the remote. If there was any issues with that, then you would definitely need someone for it. But I think if you were doing it, if you did it on a regular enough basis, if a person was having the chemo do them on a regular basis, then after about two sessions they’d be comfortable. I reckon you’re just giving it to them and they working it out.

Participant VR007

#### Setting for Using VR

Several interviewees discussed the location of VR interventions as a factor for sustained use. Participants indicated a preference for privacy, both because of security concerns and also because of uncertainty as to how they looked with the hardware on. As a result, interviewees felt that VR would not be suited for use while waiting for an appointment in an open environment.

… Also you’d got your belongings there when you’re in a room of strangers. So, I don’t know how you’d feel about your bag just sitting there with all your Medicare information and everything in it? So, I don’t know. Probably safety point of view might be an issue also.

Participant VR001

#### Multiplayer VR Apps

Interviewees were also asked to give feedback on whether they would like to use VR collaboratively while having chemotherapy. Interviewees were mixed in their views of this, in part because they were unsure how this would work. One interviewee thought that having a collaborative VR experience during chemotherapy might be beneficial for engaging carers, friends, and family waiting around during the appointment. This is important as several participants mentioned that they felt conscious of their visitors’ boredom, and in the future they would only be likely to use the VR if they were unaccompanied.

…Oh my goodness. If I had done that with one of my friends, I think it would be good. It was nice having [the researcher] there because whilst I was playing I could still hear her and my mother in law talking, so I felt like my mother in law was being entertained or I can imagine if it was my husband and I going, and I was doing this and he was just sitting there. Not that he wouldn’t have anything to do, but it’d be fun to play, two playing games or something if you could. All like challenge each other to see if you can get through the farthest.

Participant VR007

Other interviewees thought it would add another level of engagement being able to play VR apps with others, as it would add another layer of competition.

…That’d be absolutely fantastic. Well, it’s competing with another person again. So, you know, and also, well it may not even be a person there. It could be someone anywhere in the world that you’re competing with. But yes, competing against other people is always even better than competing against yourself.

Participant VR001

## Discussion

### Participant Experience

This study explored the feasibility and acceptability of using VR during chemotherapy appointments for adult patients. Patient participants reported high levels of engagement and interest in the VR intervention, the majority of whom indicated they would like to use it again for future chemotherapy sessions. Very few reported discomfort, and where it was reported, it could easily be rectified by readjusting the headset. Some patients reported VR to be a useful distraction during chemotherapy, particularly for reducing anxiety or alleviating boredom.

Although patients indicated that the VR did distract them from boredom during the chemotherapy appointment, it was interesting to note that it did not noticeably change their perception of time. Although several studies have evaluated the role of VR during chemotherapy as a distraction from anxiety and pain [16,38], there has been less research on how it influences perception of time. In one study, patients consistently underestimated the time of their chemotherapy due to the VR intervention [12]. One explanation is that our participants were aware of the duration of their chemotherapy session and used that as a guide to estimate the duration of the VR intervention. As such, time estimates are not necessarily an indicator of the acceptability of VR in this study. Other research has also suggested that coping style may affect the extent to which VR is distracting during chemotherapy [16]; however, this requires further investigation.

The intervention did not alter temporal perception, but some patients noted that it may reduce anxiety about attending an appointment. This was an unexpected finding of the study, which may warrant further research to understand the role of VR for improving treatment adherence. Patients undergoing medical treatment for cancer are known to experience anxiety [39]; however, unlike psychosocial factors such as depression, the role of anxiety on treatment adherence is unclear [40]. Nonattendance at scheduled appointments is a known burden on health care organizations [41]. Psychosocial factors are among the wide range of variables that have been identified as influencers of a patient’s likelihood not to attend a scheduled appointment [42].

Findings from this study showed that patients did not experience any significant nausea from the VR, except in instances where the lenses could not be sufficiently adjusted for the appropriate focal length. This finding addresses a recognized gap in research into the side effects of VR for patients undergoing chemotherapy [38]. Although findings from this study showed minimal adverse events from the VR intervention, the intervention was not sufficiently powered to test a reduction in side effects of treatment, such as nausea, fatigue, and pain.

Finally, based on the study results, we can provide three main recommendations for the implementation of VR for patients undergoing chemotherapy. First, additional resources (eg, volunteers) are needed to orient patients to the technology. Participants indicated that at-elbow support was preferred and that staff may not have the capacity to provide this. Second, it is important to ensure there are a range of reliable, pretested VR apps available, including both game-based and experience-based programs. Apps could support both individual and collaborative experiences that would allow visitors to play with the patient. Third, VR interventions should be conducted in private places where patients’ belongings can be securely stored.

### Limitations

A key limitation of this study is the small sample size, which limited the analyses that could be performed. Despite this, data saturation was reached for the qualitative analyses. Recruitment issues that may be of interest to other researchers in VR and chemotherapy included late cancellations due to blood tests, which precluded patients from chemotherapy at their scheduled time. In addition, due to the COVID-19 pandemic and the vulnerability of the patient participants, the researchers cancelled some scheduled sessions.

The study also used the Samsung Gear headset, which is an older type of technology that was discontinued commercially during the study. Future researchers should consider further investigating the temporal aspects of VR as a distraction for patients undergoing chemotherapy. As the findings of this study did not align with the results from broader research, it would be interesting to better understand whether cancer patients have unique experiences with VR, or whether there are characteristics of the type of treatment patients underwent that limited the ability for VR to act as an effective distraction.

Finally, we believe that this is one of the first studies to explore the effect of VR on a patient’s perception of time passing. This finding emerged from use of an observational methodology, but there would be value in attempting to replicate this study or to further explore this effect using alternative methodologies. Future researchers should consider exploring the temporal distortion effect of VR for patients undergoing chemotherapy. This could include developing a richer understanding as to why patients undergoing chemotherapy may or may not have their perception of time altered due to the use of VR during infusion, as well as the use of robust measures such as validated scales to understand the temporal effect VR has on the perception of pain, discomfort, and immersion.

### Conclusions

Patients undergoing chemotherapy enjoy having access to VR during their appointments, particularly if there is a choice of apps for them to use. It is feasible to implement VR during appointments in the controlled confines of a research study. However, sustained implementation of VR requires additional resources to provide at-elbow support for patients using the technology, to review apps for inclusion for patients, and to ensure that the VR intervention is able to be conducted in a private and secure location.

## Appendix

Multimedia Appendix 1 Postintervention survey informed by the Player Experience of Needs Satisfaction (PENS) instrument.

Multimedia Appendix 2 Adverse event checklist that allowed participants to visually indicate any adverse events experienced.

## Abbreviations

EOI: Expression of Interest
ESAS-r: Edmonton Symptom Assessment Scale revised
HMD: head-mounted display
PENS: Player Experience of Needs Satisfaction
VR: virtual reality
