# Supplementary material for: Experiences of Patients Undergoing Chemotherapy With Virtual Reality: Mixed Methods Feasibility Study
Source: JMIR Serious Games. 2022 Feb 21;10(1):e29579. doi: 10.2196/29579 (PMC8902671; doi:10.2196/29579)
Supplement: Multimedia Appendix 2 [file games_v10i1e29579_app2.pdf]

The use of an adverse event checklist that allowed participants to visually indicate any adverse events experienced.

Please mark on these pictures where it is you hurt.

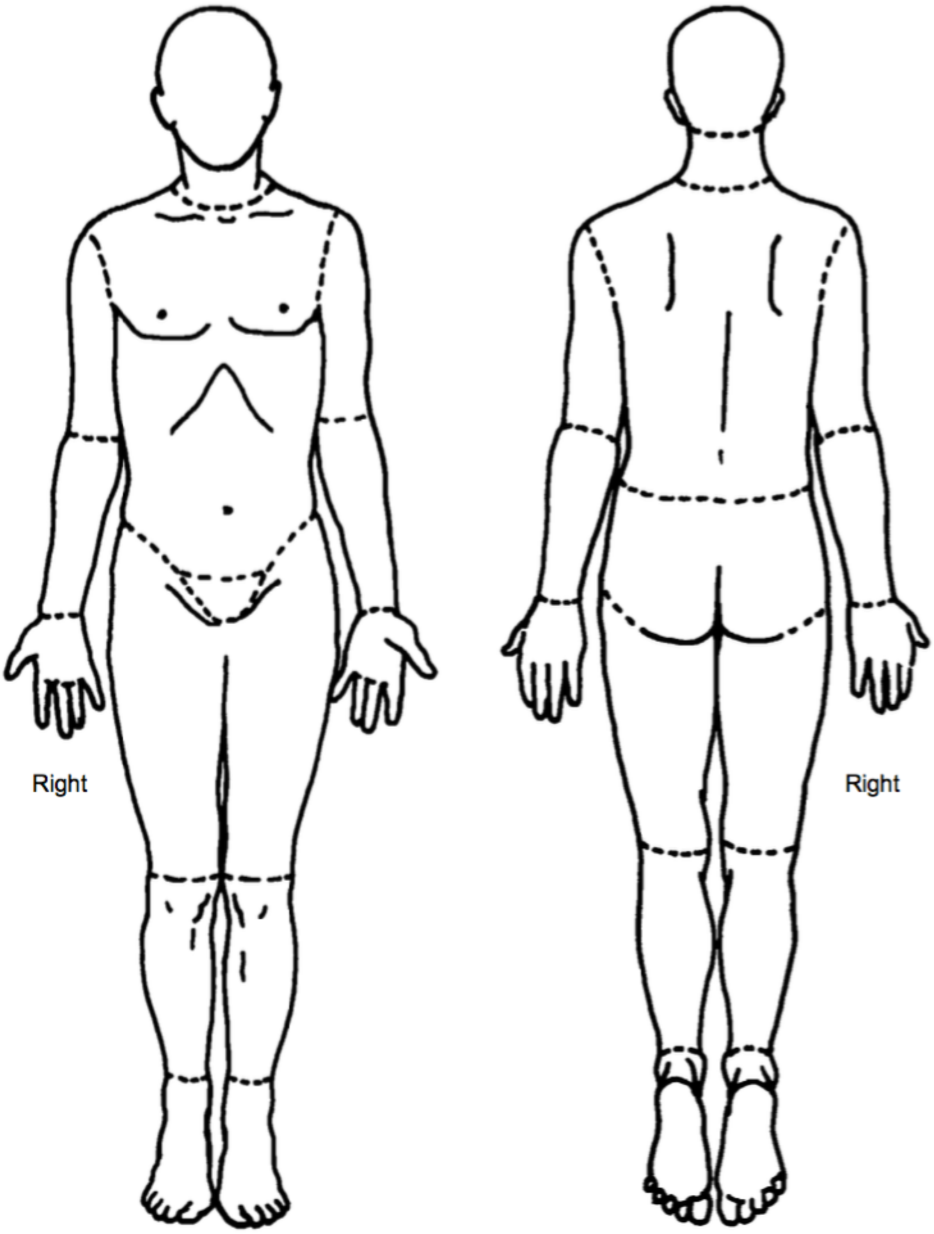

Adverse event checklist

Other information:

____

____

____
