# Supplementary material for: Experiences of Patients Undergoing Chemotherapy With Virtual Reality: Mixed Methods Feasibility Study
Source: JMIR Serious Games. 2022 Feb 21;10(1):e29579. doi: 10.2196/29579 (PMC8902671; doi:10.2196/29579)
Supplement: Multimedia Appendix 1 [file games_v10i1e29579_app1.pdf]

Post-Intervention Survey

	Strongly Disagree						Strongly Agree
**Interest/Enjoyment**	1	2	3	4	5	6	7
I enjoyed doing this activity very much.							
This activity was fun to do.							
I thought this was a boring activity.							
This activity did not hold my attention at all.							
I would describe this activity as very interesting.							
I thought this activity was quite enjoyable.							
While I was doing this activity, I was thinking about how much I enjoyed it.							
**Future Participation**	1	2	3	4	5	6	7
I would be open to trying VR again in future							
**Motion Sickness**							
Did you experience motion sickness during virtual reality session?	☐Yes ☐No						
